# Defining the diagnostic effectiveness of genes for inclusion in panels: the experience of two decades of genetic testing for hypertrophic cardiomyopathy at a single center

**DOI:** 10.1038/s41436-018-0046-0

**Published:** 2018-06-06

**Authors:** Francesco Mazzarotto, Francesca Girolami, Beatrice Boschi, Fausto Barlocco, Alessia Tomberli, Katia Baldini, Raffaele Coppini, Ilaria Tanini, Sara Bardi, Elisa Contini, Franco Cecchi, Elisabetta Pelo, Stuart A. Cook, Elisabetta Cerbai, Corrado Poggesi, Francesca Torricelli, Roddy Walsh, Iacopo Olivotto

**Affiliations:** 1 Cardiomyopathy Unit, Careggi University Hospital, Florence, Italy; 2 Cardiovascular Research Center, Royal Brompton and Harefield NHS Foundation Trust and Imperial College London, London, UK; 3 Department of Clinical and Experimental Medicine, University of Florence, Florence, Italy; 4 Genetic Unit, Careggi University Hospital, Florence, Italy; 5 Department NEUROFARBA, University of Florence, Firenze, Italy; 6 National Heart Centre, Singapore, Singapore; 7 Duke–National University of Singapore Medical School, Singapore, Singapore

**Keywords:** Diagnostic effectiveness, HCM mimics, Hypertrophic cardiomyopathy, Mendelian HCM genetics, NGS

## Abstract

**Purpose:**

Genetic testing in hypertrophic cardiomyopathy (HCM) has long relied on Sanger sequencing of sarcomeric genes. The advent of next-generation sequencing (NGS) has catalyzed routine testing of additional genes of dubious HCM-causing potential. We used 19 years of genetic testing results to define a reliable set of genes implicated in Mendelian HCM and assess the value of expanded NGS panels.

**Methods:**

We dissected genetic testing results from 1,198 single-center HCM probands and devised a widely applicable score to identify which genes yield effective results in the diagnostic setting.

**Results:**

Compared with early panels targeting only fully validated sarcomeric HCM genes, expanded NGS panels allow the prompt recognition of probands with HCM-mimicking diseases. Scoring by “diagnostic effectiveness” highlighted that *PLN* should also be routinely screened besides historically validated genes for HCM and its mimics.

**Conclusion:**

The additive value of expanded panels in HCM genetic testing lies in the systematic screening of genes associated with HCM mimics, requiring different patient management. Only variants in a limited set of genes are highly actionable and interpretable in the clinic, suggesting that larger panels offer limited additional sensitivity. A score estimating the relative effectiveness of a given gene’s inclusion in diagnostic panels is proposed.

## INTRODUCTION

Hypertrophic cardiomyopathy (*HCM*, *OMIM* 192600) is an inherited heart disease affecting approximately 1 in 500 individuals,^1^ defined by the presence of increased left ventricular wall thickness (≥15 mm in adults) that is not explained by abnormal loading conditions.^2^ Despite ever-increasing knowledge in understanding its genetic architecture, several issues remain unresolved. Reduced penetrance, allelic heterogeneity, and variable expressivity are long-known complicating factors in identifying causative variants.^3,4^ Furthermore, the existence of physiologically diverse HCM-mimicking diseases often renders differential diagnosis difficult for the cardiologist, despite a well-defined diagnostic framework for cardiomyopathies.^5,6^ Genetic testing has progressively led to identification of the pathogenic variant in up to 60% of patients, classically relying on Sanger sequencing of sarcomere genes robustly associated with HCM through family studies in large pedigrees (*MYH7*, *MYBPC3*, *TNNT2*, *TPM1*, *MYL2*, *MYL3*, *TNNI3*, and *ACTC1*).^7–9^ The advent of increasingly cost-effective next-generation sequencing (NGS) solutions has triggered a large number of candidate gene studies, often leading to gene–HCM associations based only on the identification of rare variants in patients, without controlling for the background population variation. Such genes have been subsequently included in expanded diagnostic panels, carrying the hope of reducing the proportion of affected individuals in whom the causing defect remains elusive. Following the release of the Exome Aggregation Consortium (ExAC) population database,^10^ many of the associations with HCM proposed in the absence of solid segregation studies have been critically revised and judged to be based on insufficiently stringent criteria.^11^ To contribute to the definition of a reliable core set of genes implicated in Mendelian HCM, we developed and applied a widely applicable gene- and disease-specific score (“diagnostic effectiveness” (DEff)) to estimate the relative effectiveness of a given gene in a diagnostic panel.

## MATERIALS AND METHODS

### HCM cohort

**Fig. 1:**
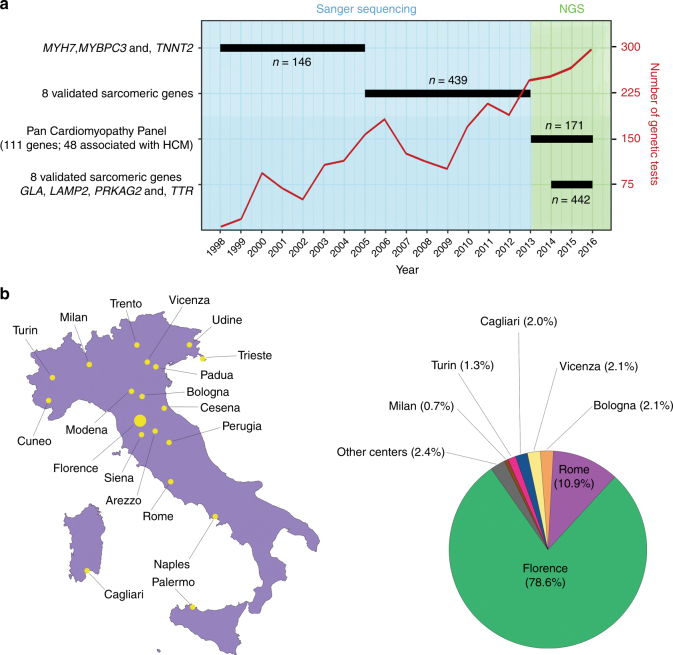
Hypertrophic cardiomyopathy cohort and gene panels. **a** Adoption of Sanger sequencing and next-generation sequencing (NGS) assays from 1998–2016, alongside the number of probands tested on each panel included in the study. The total number of genetic tests performed each year on hypertrophic cardiomyopathy (HCM) probands and their family members is also displayed (red line). The “8 validated sarcomeric genes” were *MYH7*, *MYBPC3*, *TNNT2*, *TPM1*, *MYL2*, *MYL3*, *TNNI3*, and *ACTC1*. Details of the 48 genes associated with HCM and targeted by the Pan Cardiomyopathy Panel are available in Supplementary Table S1 online. **b** Referring centers of origin for the HCM probands sequenced with NGS panels (*n* = 613) from 2013 onwards.

Careggi University Hospital is a large National Health Service facility integrated with the University of Florence. Over the past four decades, a dedicated cardiomyopathy unit has routinely evaluated and followed >1,600 patients with forms of HCM. Since 1998, all were offered genetic testing and 1,198 probands were sequenced until November 2016: 585 using Sanger sequencing of 3 or 8 sarcomeric genes and 613 using NGS of 12 or 48 genes associated with HCM (Fig. 1a). The majority of probands were originally evaluated at our center (78.5%), with the rest referred for specialist evaluation from 18 other Italian hospitals (Fig. 1b). All participants gave written informed consent and the study was approved by the relevant regional research ethics committee.

### Genetic testing

Routine genetic testing for HCM patients was introduced at our center in 1998 using Sanger sequencing and targeting the first three genes associated with HCM: *MYH7*, *MYBPC3*, and *TNNT2*.^12–14^ The set of targeted genes was expanded in 2005 to include the other five sarcomeric genes irrefutably associated with HCM (*TPM1*, *MYL2*, *MYL3*, *TNNI3*, and *ACTC1*). Following the expansion of NGS and its widespread adoption in clinical practice (fueled by its decreasing prices and escalating throughput), genetic screening by Sanger sequencing was replaced with NGS at our center in early 2013. For 1 year, all cardiomyopathy patients were screened on a 111-genes Pan Cardiomyopathy Panel, also targeting 48 genes with at least one published association with primary HCM in the literature (Supplementary Table S1 online). This gene selection rationale led to the inclusion of some genes primarily associated with RASopathies but also implicated in HCM, such as *RAF1*, and the exclusion of others not implicated in primary HCM before 2013, such as *BRAF*. In addition, as infant-onset phenotypes such as Pompe disease are assessed at the nearby Meyer University Hospital (one of the largest Italian pediatric hospitals) rather than at Careggi University Hospital, genes associated with such conditions were not included in the analysis of this cohort. From 2014, a smaller panel including only the eight validated sarcomere genes (“group α genes”) and four genes associated with HCM mimics (*GLA*, *LAMP2*, *PRKAG2*, and *TTR*—“group β genes”) was routinely adopted. Since then, the Pan Cardiomyopathy Panel (targeting group α, group β, and 36 additional genes associated with HCM defined as “group γ”) was used only on a subset of HCM patients with atypical disease features (Fig. 1a). This two-panel strategy was adopted following a diagnostic resource optimization rationale, as patients with a classic HCM phenotype were most often either genotype negative, or positive for a sarcomeric variant. Details on sequencing and bioinformatics data processing are available in Supplementary Note 1. All variants reported in this work were validated by Sanger sequencing and classified as pathogenic (P), likely pathogenic (LP), or of uncertain significance (VUS) following guidelines and standard criteria available at the time of testing. For validation purposes, all variants were also reclassified within this work according to the latest variant interpretation guidelines^15^ using CardioClassifier^16^ or InterVar^17^ (for variants in genes that CardioClassifier does not analyze). Of note, this automatic reclassification effort could not account for some guideline criteria requiring manual curation by the geneticist (details in Supplementary Note 2), as a full reanalysis of the results from 19 years of genetic testing was beyond the scope of our study.

### Derivation of DEff

The main objective of this work is to contribute to the definition of a core set of genes reliably implicated in Mendelian HCM and its mimics. To do so, we computed gene-specific parameters derived from the frequency of rare (minor allele frequency (MAF)  < 0.01%) and potentially clinically significant variants (classified as P, LP, or VUS) identified in patients, and the background population variation of rare coding variants derived from the ExAC. The MAF cutoff of 0.01% to define rarity was selected in the light of recent literature on the genetic architecture of HCM,^11,18^ considering any variant observed at higher frequencies too common to plausibly cause HCM under Mendelian dominant inheritance.

The frequency of rare and potentially clinically significant variant carriers in patients, defined for any gene as:

$$F_{{{{\rm{rare}}\,{\rm{P}},{\rm{LP}},{\rm{VUS}}}}} = \frac{{{{{\rm{Number}}\,{\rm{of}}\,{\rm{HCM}}\,{\rm{patients}}\,{\rm{with}}\, \ge 1\,{\rm{rare}}\,{\rm{P}},{\rm{LP}},\,{\rm{or}}\,{\rm{VUS}}}}}}{{{{{\rm{Number}}\,{\rm{of}}\,{\rm{HCM}}\,{\rm{patients}}}}}}$$

is suggestive of the contribution of the gene to the overall disease burden. The proportion of *F*_rare P,LP,VUS_ constituted by P/LP variant carriers (calculated without including VUS-only carriers in the numerator) reflects the actionability for cascade screening (ACS) of the variation in the gene, as VUS are not screened in family members, with the test result remaining inconclusive and not actionable. We therefore defined:

$${\rm{ACS}} = \frac{{F_{{{{\rm{rare}}\,{\rm{P}},{\rm{LP}}}}}}}{{F_{{{{\rm{rare}}\,{\rm{P}},{\rm{LP}},{\rm{VUS}}}}}}}$$

Many variants are scarcely characterized and often the only feature suggesting potential pathogenicity is their rarity in population databases, which in absence of additional evidence leads to classification as VUS.^15^ On this basis, we can assume the overall frequency of rare P/LP/VUS variants in a gene to be approximately equal to the overall frequency of rare variants:

$$F_{{{{\rm{rare}}\,{\rm{P}},{\rm{LP}},{\rm{VUS}}}}} \cong F_{\rm{{rare}}}$$

Each gene’s *F*_rare_ is also computable on population datasets such as ExAC, assuming rare variants to occur in distinct individuals and thus enabling estimation of the gene’s background population variation. Here, we compare the frequency of rare P/LP/VUS variants in HCM patients with that of rare variants in ExAC over each gene. Patients are not expected to be enriched for benign variants; therefore, a high *F*_rare_HCM:*F*_rare_ExAC ratio means that those affected carry rare variants several times more often than controls, and reflects a high likelihood for any variant in the gene in question identified in a patient to be pathogenic. This case-to-control burden ratio de facto represents the diagnostic interpretability (DI) of variation in the gene, defined in this work as:

$${\rm{DI}} = \frac{{F_{{{{\rm{rare}}\,\,{\rm{P}},{\rm{LP}},{\rm{VUS}}}\,}}{\rm{HCM}}}}{{F_{\rm{{rare}}}\,{\rm{ExAC}}}}$$

DI is influenced by both the number of rare benign variants and the penetrance of pathogenic variants in the gene in question, as genes with higher-penetrance variants and/or particularly intolerant to genetic variation will be characterized by low *F*_rare_ExAC and therefore higher DI scores.

High ACS and DI do not necessarily co-occur, as variation in a gene may be highly actionable and poorly interpretable (e.g., pathogenic variants occurring in a specific hotspot, with a high background variation rate in the rest of the gene), or vice versa (e.g., novel gene with rare and poorly characterized variants, but significantly more common in patients than controls).

Since the effectiveness of a gene when included in diagnostic panels depends on both actionability and interpretability, we define:

$${\rm{{DEff}= ACS \ast DI}}$$

$${\rm{corresponding}}\,{\rm{also}}\,{\rm{to}}\,\frac{{F_{{{{{\rm rare}}\,\,P,\,LP}}}}}{{F_{{{{{\rm rare}}}}}\,{\rm{ExAC}}}}.$$

We used DEff to measure the effectiveness of each gene when screened in HCM, and replicated DEff scores following automated variant reclassification per the latest ACMG guidelines,^15^ and on a larger published HCM cohort totaling up to 6,179 patients.^18^

### Statistical methods

All statistical analyses were performed using R. Comparisons of proportions were done by means of two-tailed Fisher's exact test, while a Mann-Whitney *U*-test was adopted to compare score distributions. A nested binomial regression model comparison by means of a likelihood ratio test was used to assess the predictivity of the genetic test date on test results.

## RESULTS

### Variation in sarcomeric genes and comparison of NGS and Sanger

In total, 1,198 probands with a diagnosis of HCM underwent genetic testing. The majority were males (63.3%) and white (98.1%), with 27.2% reporting a positive family history of HCM. Age at diagnosis (mean ± SD) was 46.3 ± 19.3 years, and patients presented with a cardiac phenotype typical of HCM, with a maximal left ventricular wall thickness of 20.5 ± 5.4 mm and left ventricular ejection fraction of 62.7 ± 10.9% (Supplementary Table S2 online). All probands were screened for variants in *MYBPC3*, *MYH7*, and *TNNT2*, and 1,052 (87.7%) were also evaluated on the other five validated HCM sarcomeric genes (*TNNI3*, *MYL2*, *MYL3*, *TPM1*, and *ACTC1*) (Fig. 1a). Of these 1,052 probands screened on all 8 group α genes, 491 (46.7%) harbored ≥1 variant classified P, LP, or VUS at the time of testing. The yield was comparable between Sanger and NGS, both in total (211 (48.1%) vs. 280 (45.7%), respectively; *P* = 0.45) and with regard to individual genes (Table 1).

**Table 1:** Proportion of P/LP/VUS variant carriers for the patient subsets screened with Sanger versus NGS, over the eight validated HCM sarcomeric genes

Gene	Variant positive (Sanger subset; *n* = 439)	Variant positive (NGS subset; *n* = 613)	Variant positive (NGS + **Sanger subsets;** ***n*** = 1,052)	***P*** value for comparison of Sanger versus NGS
*MYBPC3*	121 (27.6%)	167 (27.2%)	288 (27.4%)	1
*MYH7*	71 (16.2%)	78 (12.7%)	149 (14.2%)	0.34
*TNNT2*	12 (2.7%)	18 (2.9%)	30 (2.9%)	1
*TNNI3*	6 (1.4%)	23 (3.8%)	29 (2.8%)	0.17
*MYL2*	7 (1.6%)	5 (0.8%)	12 (1.1%)	0.51
*MYL3*	0 (0.0%)	7 (1.1%)	7 (0.7%)	0.18
*TPM1*	2 (0.5%)	4 (0.7%)	6 (0.6%)	1
*ACTC1*	1 (0.2%)	2 (0.3%)	3 (0.3%)	1

*HCM*, hypertrophic cardiomyopathy; *LP* likely pathogenic; *NGS* next-generation sequencing; *P* pathogenic; *VUS* variant of uncertain significance.

Data refer to the 1,052 probands tested on all 8 genes. Reported *P* values are adjusted for testing eight genes with the false discovery rate method.

Conversely, a significant difference was observed between the early 146 probands, tested between 1998 and 2005 only on *MYBPC3*, *MYH7*, and *TNNT2*, and the other 1,052 probands tested on all group α genes. Comparing the yield of P/LP/VUS variants in these three sarcomeric genes, the subset of probands tested early on the 3 gene panel was significantly enriched for variants (78 (53.4%) vs. 447 (42.5%); *P* = 0.02). This difference is partly explained by two distinct founder effects, causing an enrichment in Tuscan patients of otherwise rare HCM-associated variants NM_000256.3 (*MYBPC3*):c.772 G > A and NM_000257.3 (*MYH7*):c.2606 G > A. These regional founder effects were already well known to the clinical community when genetic testing started in 1998; hence, the vast majority of carriers were tested early, when the Sanger panels were in use (69/585 (11.8%) Sanger-evaluated founder variant carrier probands versus 29/613 (4.7%) NGS-evaluated probands; *P* = 7.7 × 10^−6^).

### Expanded gene set and scoring by DEff

**Fig. 2:**
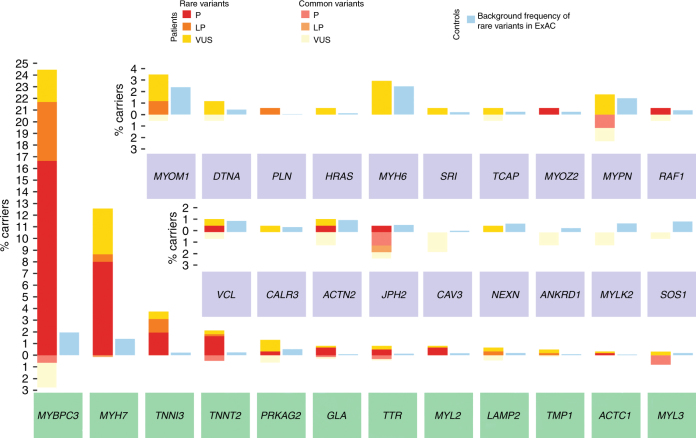
Cumulative variant frequencies observed in hypertrophic cardiomyopathy patients compared with those in the Exome Aggregation Consortium. Proportion of hypertrophic cardiomyopathy (HCM) probands evaluated by next-generation sequencing (*n* = 613) carrying clinically reportable variants (left-hand side bar for each gene). Proportions are divided into “rare” (minor allele frequency (MAF) < 0.01% in the Exome Aggregation Consortium (ExAC); above 0 on the *y* axis) and “common” (MAF > 0.01% in ExAC; below 0 on the *y* axis). Variants with MAF > 0.01% are unlikely to be truly disease causing, as recently demonstrated.^26^ Variation in HCM is also divided into pathogenic (P: red, rare; pale red, common), likely pathogenic (LP: orange, rare; pale orange, common), and variants of uncertain significance (VUS: yellow, rare; pale yellow, common). Light blue bars (right-hand side for each gene) represent the proportion of rare protein-altering variant carriers in ExAC. Robustly validated disease genes (turquoise background) were sequenced in all probands screened using next-generation sequencing (*n* = 613), while other genes (purple background) were sequenced on a subset of 171 probands (Fig. 1). Genes associated with syndromic forms of HCM are indicated in bold. For example, *MYBPC3* is characterized by features typical of a major disease gene: strong rare variation excess in patients (left-hand side bar) versus ExAC (right-hand side bar), reflective of high diagnostic interpretability, and a high proportion of P/LP variants (red/orange), indicating high actionability for cascade screening (see Materials and Methods for derivation). Conversely, *MYPN* is characterized by all variants originally classified as P/LP being too common in ExAC to be pathogenic, the frequency of rare variant carrier patients being essentially equal to the background population level (low diagnostic interpretability), and all rare variants in patients being VUS (null actionability for cascade screening)

As the adoption of NGS from 2013 allowed the systematic screening of a higher number of genes, the results obtained on the 613 NGS-screened probands were analyzed in detail and used to compute all gene-specific scores proposed in this work. In doing so, we dissected variation by gene, diagnostic classification, and rarity, and compared cumulative rare variant frequencies observed in HCM patients with those in ExAC (Fig. 2). The full set of analyzed variants, alongside their frequency and diagnostic interpretation details, is available in Supplementary Table S3 online.

**Fig. 3:**
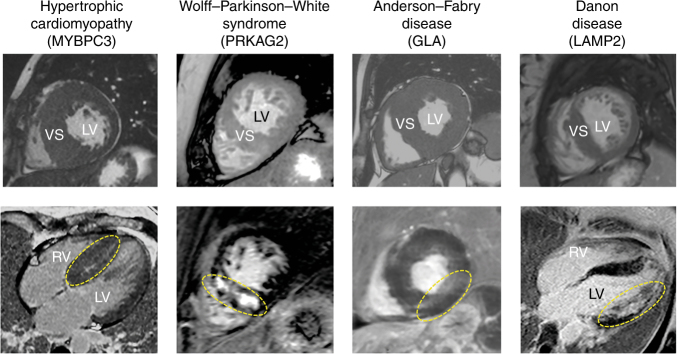
Cardiac magnetic resonance images of example patients with sarcomeric hypertrophic cardiomyopathy and three of its mimics identified in our cohort. Top: cine two-dimensional images in short-axis view using balanced turbo-field echo sequencing. Bottom: late gadolinium enhancement patterns (circled in yellow) in four-chamber (first and last panel) and short-axis view, using spoiled inversion recuperation turbo-field echo three-dimensional sequencing. *LV* left ventricle; *RV* right ventricle; *VS* ventricular septum

Variants originally classified P/LP/VUS were detected in 31 of 48 HCM-associated genes targeted by NGS panels. Nineteen probands (3.1%) were diagnosed with metabolic or infiltrative forms closely mimicking sarcomeric HCM (Fig. 3), including transthyretin-related amyloidosis (*n* = 6, 1%), Fabry disease (*n* = 5, 0.8%), Danon disease (*n* = 4, 0.7%) and *PRKAG2*-related HCM (*n* = 4, 0.7%), following the identification of variants in group β genes.

Of note, only rare variants (MAF < 0.01% in ExAC) were considered in the derivation of the gene-specific scores proposed. This was to minimize the effect of false positive P/LP variant classifications and enhance replication on published cohorts where variants with this MAF were reported.

**Fig. 4:**
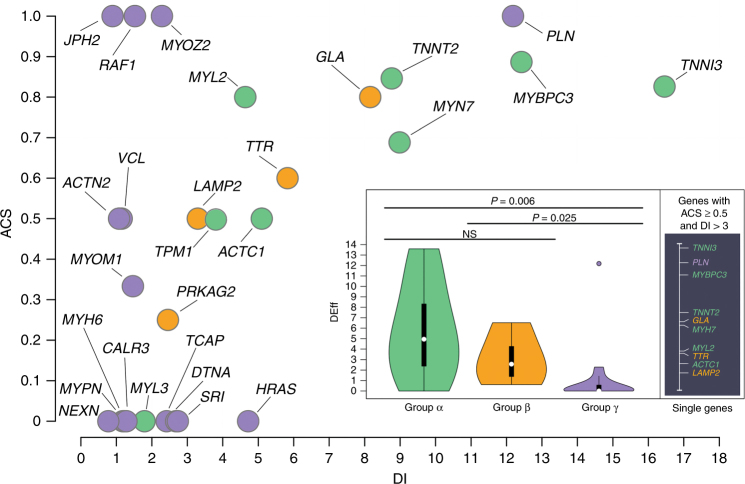
Group α, β, and γ genes characterized by overall actionability for cascade screening and diagnostic interpretability ratios. Per-gene diagnostic interpretability (DI) versus actionability for cascade screening (ACS). DI describes the relative frequency of potentially pathogenic variants in patients compared with controls corresponding, in the diagnostic setting, to the odds of a variant detected in a patient to be causative (e.g., based on our data, a potentially pathogenic variant detected in *MYH7* in a patient is approximately nine times more likely to be causative than benign). ACS reflects the proportion of potentially pathogenic variants detected in patients that are actionable for cascade screening family members (e.g., approximately 70% for *MYH7*). The product of the two metrics yields the diagnostic effectiveness (DEff) of each gene (inset; see Materials and Methods), which summarizes the relative utility for inclusion of a given gene in a diagnostic panel and is significantly different for groups α and β versus group γ genes. Notably, the only group γ gene with a DEff comparable to scores of fully validated group α and β genes is *PLN*. Single DEff values of representative genes with high values of ACS and DI are displayed to the right of the inset, where *TPM1* (DEff = 1.9) was excluded for improved readability. NS, not significant

Group α, β, and γ genes were characterized by overall ACS (i.e., the proportion of rare P/LP variants of the total rare P/LP/VUS variation in the gene in patients; median (interquartile range): 0.74 (0.5–0.83), 0.55 (0.44–0.65), and 0 (0–0.75), respectively) and DI ratios (i.e., the ratio of rare, potentially pathogenic variants observed in patients versus ExAC controls; 7.2 (5.0–9.8), 4.6 (3.1–6.4), and 1.5 (1.1–2.5), respectively) (Fig. 4).

The DEff (i.e., the gene’s ACS multiplied by its DI) of group α genes was comparable to that of group β (*P* = 0.37), but both these gene sets had values significantly higher than group γ (*P* = 0.006 and *P* = 0.025, respectively; α: 4.9 (2.4–8.3), β: 2.6 (1.4–4.3), γ: 0 (0–0.7); Fig. 4). These results were replicated when all variants were classified according to the most recent interpretation guidelines^15^—although some criteria requiring manual curation were excluded (see Materials and Methods and Supplementary Note 2)—with groups α and β characterized by comparable DEff values (*P* = 1), which were higher than those of group γ genes (*P* = 0.006 and *P* = 0.02, respectively).

An exception in group γ is *PLN* (DEff = 12.2, independent of adopted guidelines), which is rarely mutated but characterized by high actionability and interpretability. Replicated DEff, computed for a published cohort of 6,179 patients,^18^ showed group α and β values to be comparable to those obtained in our data (*P* = 0.96 and *P* = 0.56, respectively). A formal comparison of group γ DEff value distribution was not performed as only four variant-positive group γ genes were sequenced in the replication cohort (*ACTN2*, *MYOZ2*, *NEXN*, and *PLN*). While the replication DEff of *ACTN2*, *MYOZ2*, and *NEXN* was 0, *PLN*’s replication DEff score was 1.9, which is lower than its discovery value but in line with that of fully validated group α and β genes in our data. This makes it an outlier, being over 1.5 interquartile range points above the third quartile with respect to group γ (Fig. 4). All discovery and replication DEff values are provided in Supplementary Table S4 online.

Of the eight variants in group γ genes originally classified P/LP (following variant interpretation guidelines available at the time;^11^ Supplementary Table S5 online), only three (in *PLN*, *ACTN2*, and *MYOZ2*) are considered LP by the most recent guidelines.^15^ As far as variants’ population frequency is concerned, we observed a significant enrichment in common variants (MAF > 0.01%) in group γ genes compared with group α and β genes (24/41 (58.5%) vs. 38/342 (11.1%) variants, respectively; *P* = 3.0 × 10^−11^).

Finally, the genetic test date was predictive of the test result, independent of the referring hospital, gene panel, presence of founder effect variants, and variant interpretation guidelines applied (*P* = 0.02), with less recent tests characterized by a higher likelihood of yielding a positive result (P/LP/VUS identified).

## DISCUSSION

Since 1989, when HCM was first mapped to a locus on chromosome 14 and *MYH7* was robustly associated with disease for the first time,^12^ variants in 7 other genes encoding proteins of the cardiac sarcomere (*MYBPC3*, *TNNT2*, *TPM1*, *MYL2*, *MYL3*, *TNNI3*, and *ACTC1*) have been indisputably linked to HCM ^7^ by multiple linkage studies in large pedigrees. Genetic testing is historically reported to identify a disease-causing variant in 40–60% of patients fulfilling HCM diagnostic criteria, with a large predominance of pathogenic variants in these eight genes (group α).^8,9^ Consistently, we found ≥1 variant of clinical significance (VUS, LP, or P) in group α genes in 491 (46.7%) probands screened on all 8 genes. Phenotypic and demographic characteristics of the analyzed HCM cohort resemble those of the typical HCM patient group in an Italian hospital, with a male-to-female ratio close to 3:2 and a predominance of Caucasian individuals.

While Sanger sequencing and NGS pose radical differences—with NGS characterized by a lower per-base cost and higher sequencing throughput—it has been established that the two techniques are characterized by comparable accuracy. In line with previously published reports,^19,20^ we did not observe significant yield discrepancies over the 8 group α genes between Sanger sequencing (*n* = 585 patients) and NGS (*n* = 613 patients).

Given the many dubious associations of nonsarcomeric genes with HCM that have been proposed since 1999,^21^ we devised the novel gene- and disease-specific DEff to measure the utility for inclusion of a given gene in a panel. DEff summarizes information on a gene's potential for family cascade screening and the effective likelihood of pathogenicity of its variation when found in affected patients, with the underlying assumption that the DEff scores observed for fully validated disease genes represent the golden standard for inclusion in diagnostic panels. Not surprisingly, DEff scores were significantly higher overall for group α and β genes associated with HCM or its mimics based on complete and irrefutable evidence, compared with group γ genes. Approximately 71% of probands screened with NGS (*n* = 436) underwent genetic testing before publication of the latest variant interpretation guidelines;^15^ therefore, retaining the original variant classification represents a limitation of this work. To overcome this, we performed automated reclassification of all variants using CardioClassifier^16^ and InterVar,^17^ but without the possibility of evaluating parameters requiring manual curation by the geneticist (see Materials and Methods and Supplementary Note 2). Nonetheless, this represents a conservative evaluation of variants’ pathogenicity, underrating the disease-causing potential of group α and β genes especially (for which there is more published segregation and functional evidence). However, using these updated and conservative classifications, the DEff scores of groups α and β were again observed to be significantly higher than group γ, underscoring the reliability of DEff.

Within group γ, *PLN* was an upper outlier in terms of its DEff score (a result replicated for a larger HCM cohort of 2,167 patients). Thus, although the low frequency of *PLN* variants has understandably hampered the availability of large affected families with a clear HCM phenotype to prove co-segregation, we suggest *PLN* for routine incorporation in diagnostic gene panels for HCM. As DEff is intrinsically based on observed variation, we did not calculate it for the variant-negative genes (all in group γ). As such—and as the number of patients sequenced on the Pan Cardiomyopathy Panel was relatively low (*n* = 171)—we acknowledge that if any group γ gene has a rare pathogenic role, it could have remained undetected in this analysis (especially for genes that were not sequenced in the replication cohort). Therefore, replication of gene-specific metrics for such genes in large cohorts is warranted, although it has previously been observed that, for most of these genes, there is little or no evidence for a causative role in HCM.^11,22^ Despite the reduced size of the subset with sequenced group γ genes, the remaining 18 variant-positive γ genes performed poorly in terms of actionability and/or interpretability, underscoring the need for sole inclusion in diagnostic panels of those genes associated with disease with rigorous evidence. Particular cases are *ACTN2* and *MYOZ2*, which are not enriched for rare variants in HCM either in our cohort (*P* = 0.12 and *P* = 0.36 versus ExAC, respectively) or larger published clinical case series,^18,21^ but with the same variants detected in our patients reported to co-segregate with HCM in large pedigrees.^23,24^ In such cases, allele-specific genetic testing is informative, as opposed to screening for variants over the complete coding sequence, with the majority of variants in patients likely to represent harmless background variation. The fact that the original P/LP classification held with updated guidelines for only three group γ variants suggests that the DEff of group γ genes computed here represents a conservative overestimate, and highlights the importance of a periodic revision of identified variants’ pathogenicity classification in diagnostic laboratories. These findings support the idea that inclusion of most γ genes in HCM diagnostic panels lacks utility, yielding uninterpretable and unactionable results. Per current variant interpretation guidelines,^15^ most rare variants in such poorly validated genes would be considered VUS, causing further uncertainty for patients, with the risk of subjective and incorrect interpretation of an inconclusive result.^25^

One of the features considered as strong evidence for a variant being benign by current guidelines is the MAF being too high for the disease in question.^15^ Data from projects such as 1000 Genomes, the Exome Sequencing Project, and especially ExAC^10^ have demonstrated how many variants previously deemed to be pathogenic are present at population frequencies incompatibly high with Mendelian dominant disease causation. This also affects variation in validated HCM genes in which we observed 38/342 (11.1%) variants with a MAF > 0.01%, most likely representing benign bystanders of other disease causes. However, compared with the 12 validated group α and β genes, group γ genes are strongly enriched in common variants (*P* = 3.0 × 10^–11^), providing additional evidence against their HCM-causing potential. After completion of this work, more stringent, variant-specific allele frequency adjustment has been proposed^26^—with 0.004% estimated as the maximum credible allele frequency for any HCM-causing variant. Such a strategy also controls for differences between ethnic groups, correcting each variant’s MAF based on the highest observed in the different ethnic subgroups. The application of such a valid and innovative framework in our case affected only 7 variants (in 5 different genes and all carried by a single patient) of the 393 detected, which were potentially too common to cause HCM (Supplementary Table S6 online), thus not impacting our results. Of note, all these variants are classified VUS by current guidelines,^15^ and only 3 have a MAF > 0.01% in specific ethnic groups, suggesting that the small proportion of non-Caucasian cases included (<2%) and the usage of the entire ExAC dataset did not affect our conclusions.

Finally, the observation of diagnostic yield dropping over time independent of potential confounders, as highlighted by multivariate regression modeling, is in line with previously published reports in the context of cardiomyopathies and arrhythmias.^22,27^

In conclusion, our data highlight that only genes characterized by incontrovertible evidence of an association with HCM or its metabolic mimics yield interpretable and actionable results in the diagnostic setting, with equal results obtained with Sanger sequencing and NGS. In our case, the specific advantage of an expanded gene panel was the possibility to promptly identify 19 patients (3.1%) affected with HCM mimics through a “genotype-first” approach, enabling early diagnosis of these conditions—sometimes indistinguishable from classic HCM in the clinic—and requiring radically different patient management. Such a change in clinical management represents additional actionability—on top of that represented by cascade screening in families—characterizing genes associated with metabolic or infiltrative HCM-mimicking disease. The adoption of NGS thus allowed a small set of additional important genes to be routinely screened at little extra cost, with HCM mimics previously diagnosed at our center through traditional laboratory assays following specialist clinical assessment, in which the presence of the HCM-mimicking disease was at least suspected. Recognizing such conditions in the initial stages is key, given the better response to treatment of Fabry patients when enzyme replacement therapy is started earlier,^28^ and given that sudden cardiac death is a frequent cause of death in Danon disease.^29^ Through the newly devised metric DEff, we suggest that *PLN* should be treated as a “core”—although rare—HCM gene, whereas for *ACTN2* and *MYOZ2* only specific screens for plausibly pathogenic variants would be effective.

DEff is widely applicable in the context of Mendelian diseases, and we believe it will be particularly useful for genetically heterogeneous conditions such as HCM for which the number of genes irrefutably associated with disease constitutes only a small proportion of all implicated genes. Such diseases can be problematic in the diagnostic setting when it comes to choosing which genes to include in panels. On the one hand, screening for only fully validated genes may cause the number of false negative tests to increase, while on the other hand, being overly inclusive and screening all implicated genes would cause an increase of inconclusive—and even false positive—findings. Both scenarios represent situations that are detrimental for patients and their family members. In this respect, DEff is intended as a score that can aid geneticists in performing a balanced selection of genes to be included in diagnostic panels, and that can be considered as additional evidence by gene curation efforts such as ClinGen,^30^ created to overcome such ambiguities.

As a whole, these data support the concept that screening a large number of genes offers limited additional sensitivity in HCM, and that novel approaches to investigate its underlying complex—and most likely not only familial^31,32^—etiological background should be developed.

## Electronic supplementary material

Supplementary Tables

Supplementary Note 1
