# Supplementary material for: Defining the diagnostic effectiveness of genes for inclusion in panels: the experience of two decades of genetic testing for hypertrophic cardiomyopathy at a single center
Source: Genet Med. 2018 Jun 6;21(2):284–92. doi: 10.1038/s41436-018-0046-0 (PMC6752309; doi:10.1038/s41436-018-0046-0)
Supplement: Supplementary file 2 — Supplementary Note 1 [file 41436_2018_46_MOESM2_ESM.docx]

# SUPPLEMENTARY NOTE 1

Automated Sanger sequencing was performed using an ABI Prism 3100 Genetic Analyzer (Applied Biosystems, Foster City, KS). Next-generation sequencing runs on the pan-cardio panel were performed using Illumina HiScan and NextSeq platforms, following library preparation with Nextera Rapid Capture Custom (Illumina, San Diego, CA) and TruSeq Custom Amplicon (Agilent, Santa Clara, CA) enrichment kits. Runs on the 12-genes panel were performed with an Illumina MiSeq sequencing platform, following library preparation with HaloPlex custom enrichment kit (Agilent, Santa Clara, CA). The bioinformatics processing of raw sequencing data included FastQC^1^ for quality-checking reads, prior to alignment to the reference human genome hg19 using BWA-MEM^2^. Duplicate reads were marked with Picard ^3^ and alignment metrics were computed with SAMtools^4^. The GATK software^5^ was used for detecting variants, prior to annotation with ANNOVAR^6^.

# SUPPLEMENTARY NOTE 2

All variants detected in the subset of patients screened with NGS (N=613) were automatically re-classified as per most recent ACMG variant interpretation guidelines^7^ using CardioClassifier^8^ or InterVar^9^. CardioClassifier is a semi-automated decision-support tool to assign a diagnostic classification to genetic variants in the context of inherited cardiac conditions. InterVar is instead a more generic classifier, generating automated interpretation on 18 guidelines criteria. CardioClassifier is not yet parameterized for all genes involved in inherited cardiac conditions, but given its superior performance in the context of cardiac conditions such as HCM, we used it whenever possible (i.e. on all variants in *ACTC1, GLA, LAMP2, MYBPC3, MYH7, MYL2, MYL3, PLN, PRKAG2, TNNI3, TNNT2* and *TPM1*). InterVar was used to re-classify a minority of variants (58 of 393) in genes not analyzed by CardioClassifier as of February 2018 (*ACTN2, ANKRD1, CALR3, CAV3, DTNA, HRAS, JPH2, MYH6, MYLK2, MYOM1, MYOZ2, MYPN, NEXN, RAF1, SOS1, SRI, TCAP, TTR* and *VCL*). Some parameters (namely, PP1 on evidence of co-segregation with disease, PM6/PS2 on *de novo* variant occurrence in association with disease and PS3/BS3 on functional data) are not automatically evaluated by CardioClassifier for the majority of variants, and require manual curation to evaluate whether they should be activated. For some known disease variants, CardioClassifier curators inserted manual evidence in the tool’s knowledgebase. Given that our re-classification effort was aimed at a validation of the *diagnostic effectiveness* with updated ACMG guidelines, we decided to remove such evidence from the tool’s classification results to make the re-classification as consistent as possible across genes. Of note, ignoring CardioClassifier’s knowledgebase evidence caused a downgrade of only one variant allele (p.R186Q in *TNNI3*) from P/LP to VUS. As to population frequency criteria (particularly BS1/BA1-PM2), CardioClassifier applies the variant-specific framework proposed by Whiffin et al.^10^. As shown in Table S6 and described in the text, only 7 variant of the 393 we analyzed would be considered too common to cause HCM by such framework, not impacting results in our case. For consistency, we have considered 0.01% as the maximum possible MAF for an HCM-causing variant also in the re-classification process (modifying the BS1/PM2 criteria for these 7 variants). However, we believe further studies should rely on the novel framework proposed by Whiffin and colleagues for variant frequency filtering.

Also InterVar does not automatically evaluate all ACMG guideline parameters when performing variant classification, leaving the user with the possibility of performing manual variant adjustment after the initial classification step. Consistently with the decision we took with CardioClassifier, we report variants’ classifications as from the criteria automatically evaluated by the tool. While InterVar activates some of the criteria that CardioClassifier does not evaluate automatically, only one variant (p.M228T in *ACTN2*) achieves LP classification thanks to one of such criteria (PM1), not significantly impacting results (and supporting the lack of clinically actionable variants in not-fully-validated disease genes).

# REFERENCES

1. Babraham Bioinformatics - FastQC A Quality Control tool for High Throughput Sequence Data. http://www.bioinformatics.babraham.ac.uk/projects/fastqc/ (4 December 2015)

2. Li H, Durbin R. Fast and accurate short read alignment with Burrows-Wheeler transform. *Bioinforma Oxf Engl* 2009;**25**:1754–1760.

3. Picard Tools - By Broad Institute. http://broadinstitute.github.io/picard/ (4 December 2015)

4. Li H, Handsaker B, Wysoker A, Fennell T, Ruan J, Homer N, Marth G, Abecasis G, Durbin R. The Sequence Alignment/Map format and SAMtools. *Bioinformatics* 2009;**25**:2078–2079.

5. McKenna A, Hanna M, Banks E, Sivachenko A, Cibulskis K, Kernytsky A, Garimella K, Altshuler D, Gabriel S, Daly M, DePristo MA. The Genome Analysis Toolkit: A MapReduce framework for analyzing next-generation DNA sequencing data. *Genome Res* 2010;**20**:1297–1303.

6. Wang K, Li M, Hakonarson H. ANNOVAR: functional annotation of genetic variants from high-throughput sequencing data. *Nucleic Acids Res* 2010;**38**:e164–e164.

7. Richards S, Aziz N, Bale S, Bick D, Das S, Gastier-Foster J, Grody WW, Hegde M, Lyon E, Spector E, Voelkerding K, Rehm HL. Standards and Guidelines for the Interpretation of Sequence Variants: A Joint Consensus Recommendation of the American College of Medical Genetics and Genomics and the Association for Molecular Pathology. *Genet Med Off J Am Coll Med Genet* 2015;**17**:405–424.

8. Whiffin N, Walsh R, Govind R, Edwards M, Ahmad M, Zhang X, Tayal U, Buchan R, Midwinter W, Wilk AE, Najgebauer H, Francis C, Wilkinson S, Monk T, Brett L, O’Regan DP, Prasad SK, Morris-Rosendahl DJ, Barton PJR, Edwards E, Ware JS, Cook SA. CardioClassifier: disease- and gene-specific computational decision support for clinical genome interpretation. *Genet Med* 2018;

9. Li Q, Wang K. InterVar: Clinical Interpretation of Genetic Variants by the 2015 ACMG-AMP Guidelines. *Am J Hum Genet* 2017;**100**:267–280.

10. Whiffin N, Minikel E, Walsh R, O’Donnell-Luria AH, Karczewski K, Ing AY, Barton PJR, Funke B, Cook SA, MacArthur D, Ware JS. Using high-resolution variant frequencies to empower clinical genome interpretation. *Genet Med* 2017;
